# Design of a Multi-Epitope Vaccine Against Ovine *Pasteurella multocida* Using Immunoinformatics Strategies

**DOI:** 10.3390/microorganisms14030656

**Published:** 2026-03-13

**Authors:** Yanjie Qiao, Aodi Wu, Honghuan Li, Youquan Zhuang, Qiang Fu, Li Yang, Huijun Shi

**Affiliations:** 1 College of Animal Medicine, Xinjiang Agricultural University, Urumqi 830052, China; 18899596250@163.com (Y.Q.); 13667632221@163.com (Y.Z.); fq198505@gmail.com (Q.F.); 2 College of Animal Science and Technology, Shihezi University, Shihezi 832003, China; 15739592133@163.com; 3 School of Medicine, Shihezi University, Shihezi 832003, China; lhh121004@126.com

**Keywords:** *Pasteurella multocida*, membrane proteins, MEV, molecular docking, immunoinformatics

## Abstract

This study aimed to design a multi-epitope vaccine (MEV) against *Pasteurella multocida* (Pm) using immunoinformatics approaches. Based on four conserved outer membrane proteins (OmpA; OmpH; PlpEand LolA), 15 immunodominant epitopes were identified, including 8 CTL epitopes, 3 HTL epitopes, and 4 B-cell epitopes. A vaccine construct was developed by incorporating RGD and PADRE adjuvant sequences. Computational analyses indicated that the vaccine possesses favorable physicochemical properties and structural stability. The molecular docking and normal mode analyses reveal a potential binding interface between the basis and TLR2/TLR4, with a computed binding energy of −10.1 kcal/mol for TLR4, suggesting a possible preferential interaction. Immune simulation predicted the vaccine could effectively elicit responses from B cells, T cells, and key cytokines such as IFN-γ. Additionally, the vaccine sequence was successfully cloned into the pET-28a (+) expression vector, facilitating future recombinant expression. This study provides a theoretical foundation for developing a safe and effective subunit vaccine against Pm.

## 1. Introduction

*Pasteurella multocida* (Pm) is a Gram-negative bacterium responsible for hemorrhagic septicemia and infectious pneumonia in sheep [1]. In addition to reducing production performance, Pm infection inflicts substantial economic losses on the sheep industry. Epidemiological studies indicate that capsular serotypes A and B are predominant in Chinese sheep herds. However, recent epidemiological data indicate that serotype D infections have been reported in regions such as Xinjiang, Inner Mongolia, and Tibet in China, with localized infection rates reaching 5–10% [2]. As an opportunistic pathogen, Pm typically colonizes the respiratory or digestive tracts and remains asymptomatic in immunocompetent hosts; however, it can trigger severe infection under stress conditions, such as transport, secondary infection, or sudden environmental changes. With the intensification of sheep farming, the widespread use of antibiotics has exacerbated antimicrobial resistance in Pm, severely complicating disease control efforts.

Vaccination remains the most cost-effective strategy for controlling Pm infection; however, current vaccine platforms possess significant limitations. Although inactivated vaccines offer a high safety profile, they provide short-term immunity and lack cross-protection. Conversely, while live attenuated vaccines induce potent immune responses, they are associated with technical challenges, including long production cycles and the risk of virulence reversion [3]. Furthermore, Pm frequently co-infects with *Mannheimia haemolytica* (Mh) or other serotypes, limiting the efficacy of existing vaccines. Consequently, there is an urgent need to screen for dominant immunogenic antigens to facilitate the development of efficient multivalent vaccines.

Multi-epitope vaccines (MEVs) have been extensively investigated in various pathogens, including *Escherichia coli* [4], *Salmonella* [5], *Klebsiella pneumoniae* [6], *Streptococcus pneumoniae* [7], *Shigella* [8], and *Brucella* [9]. Despite this, there are currently no reports on MEVs specifically targeting ovine Pm infections. Epitopes, or antigenic determinants, are specific structural targets recognized by immune cells and serve as the fundamental basis for eliciting specific immune responses. By incorporating multiple dominant B-cell and T-cell epitopes, MEVs can overcome the limitations of single-antigen vaccines, such as poor antigenicity and restricted protective efficacy. They effectively stimulate both cellular and humoral immunity [10], while minimizing allergenicity and toxicity [11].

Historically, Pm capsular antigens served as the initial candidates for subunit vaccines against pasteurellosis. Subsequently, research expanded to include various virulence factors, such as lipopolysaccharides (LPS), *Pasteurella multocida* toxin (PMT), bacteriocin-like toxins, outer membrane proteins (OMPs), and adhesins [12]. Among these, OmpA is a highly conserved integral component of the outer membrane [13]. It plays a crucial role in host cell adhesion and virulence, exhibiting both antigenicity [14]. OmpH is another major surface-exposed and conserved OMP; given its presence in nearly all clinical Pm isolates [15], it holds significant potential as a vaccine candidate [16]. PlpE, one of the most extensively studied OMPs, demonstrates superior antigenicity and cross-protective capacity, making it a critical protective antigen for Pm. Notably, in a study by Hatfaludi et al. involving 71 recombinant Pm proteins, only PlpE conferred protection in poultry [17]. Finally, the LolA protein functions both as an outer membrane component and part of the ABC transporter system [18]. It is associated with antimicrobial resistance and plays a pivotal role in pathogenicity by regulating bacterial adhesion [19]. The antigenicity of LolA has also been confirmed in other bacterial species [20].

**Figure 1:**
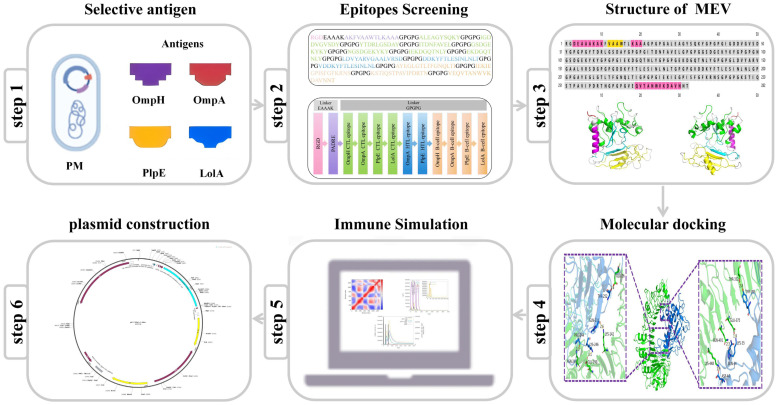
Flowchart illustrating the construction process of the multi-epitope-based peptide vaccine.

Although multi-epitope vaccine design has been widely applied in human medicine and some livestock pathogens, studies focusing on ovine Pm remain scarce in the literature. Given the significant economic impact of pasteurellosis on the sheep industry and the limitations of current conventional vaccines, there is an urgent need to explore new prophylactic strategies. In this study, we employed an immunoinformatics pipeline to design a multi-epitope vaccine specifically targeting ovine Pm, aiming to provide a theoretical basis for the development of a safe and effective vaccine against this understudied pathogen (Figure 1). This study addresses the urgent demands of the livestock industry and provides a new technical strategy for the prevention and control of animal diseases.

## 2. Materials and Methods

### 2.1. Amino Acid Sequences of the Selected Target Antigens

The amino acid sequences of the *Pasteurella multocida* dominant antigens (OmpH; OmpA; PlpE and LolA) were retrieved from the NCBI database (https://www.ncbi.nlm.nih.gov/, accessed on 8 January 2026). The antigenicity of these four proteins was subsequently predicted using the VaxiJen v2.0 server (https://www.ddg-pharmfac.net/vaxijen/VaxiJen/VaxiJen.html, accessed on 8 January 2026), with the threshold set at >0.5.

### 2.2. Prediction of B-Cell Epitopes

B-cell epitopes of the dominant antigens were predicted using the ABCpred server (https://webs.iiitd.edu.in/raghava/abcpred/index.html, accessed on 8 January 2026). The prediction parameters were set to an epitope length of 16 amino acids and a threshold of 0.88, where higher scores indicated a higher probability of the peptide serving as an immunodominant B-cell epitope.

### 2.3. Prediction of CTL Epitopes

Cytotoxic T Lymphocyte (CTL) epitopes of the dominant antigens were predicted using the IEDB MHC-I binding server (http://tools.iedb.org/mhci/, accessed on 8 January 2026). The prediction utilized the “IEDB recommended 2020.09 (NetMHCpan EL 4.1)” method targeting the HLA-A*01:01 allele with an epitope length of 9–10 amino acids. Candidates with a percentile rank < 0.5 were screened for further analysis. Subsequently, the antigenicity of the selected epitopes was evaluated using the VaxiJen v2.0 server with a threshold of 0.5. Only epitopes exhibiting an antigenicity score > 0.5 were selected as the immunodominant CTL epitopes for the construction of Pm-MEV. Due to the limited availability of ovine leukocyte antigen (OLA) allele data in public databases and epitope prediction tools, a panel of common human HLA class I alleles was used for CTL epitope prediction. A similar approach was applied for helper T lymphocyte (HTL) epitope prediction using HLA class II alleles, as described in the following section.

### 2.4. Prediction of HTL Epitopes

Helper T Lymphocyte (HTL) epitopes of the dominant antigens were predicted using the IEDB MHC-II binding server (http://tools.iedb.org/mhcii/, accessed on 8 January 2026). The prediction utilized the “IEDB recommended 2023.05 (NetMHCIIpan EL 4.1)” method targeting the HLA-DRB1*01:01 allele, with an epitope length of 15 amino acids. Epitopes with a percentile rank < 0.5 were initially selected. Subsequently, the antigenicity of these candidates was evaluated using VaxiJen v2.0 with a threshold set to 0.5; only epitopes with an antigenicity score > 0.5 were retained. Furthermore, the IFN-γ epitope server (https://webs.iiitd.edu.in/raghava/ifnepitope/application.php, accessed on 8 January 2026) was employed to assess the IFN-γ-inducing capability of the peptides. Finally, epitopes predicted to be IFN-γ positive were selected as the immunodominant HTL epitopes for the construction of Pm-MEV.

### 2.5. Construction of Pm-MEV

The predicted and screened CTL, HTL, and B-cell epitopes were fused using GPGPG linkers. To enhance antigenicity, the RGD peptide and the PADRE sequence were linked to the N-terminus of the vaccine construct via EAAAK linkers. These modifications were incorporated to ensure robust neutralizing antibody titers and to promote the amplification and persistence of the desired T helper 1 (Th1) immune response.

### 2.6. Evaluation of Physicochemical Properties of Pm-MEV

The antigenicity of Pm-MEV was evaluated using the VaxiJen v2.0 server and the IEDB Class I Immunogenicity server (https://tools.iedb.org/immunogenicity/, accessed on 9 January 2026), respectively. Physicochemical properties were characterized using the ProtParam server (https://www.expasy.org/resources/protparam, accessed on 9 January 2026), while solubility was predicted via the SOLpro server (http://scratch.proteomics.ics.uci.edu/, accessed on 9 January 2026). Additionally, the allergenicity and toxicity of the vaccine construct were assessed using the AllergenFP v.1.0 (http://ddg-pharmfac.net/AllergenFP/, accessed on 9 January 2026) and ToxinPred (https://webs.iiitd.edu.in/raghava/toxinpred/index.html, accessed on 9 January 2026) servers.

### 2.7. Prediction and Validation of Secondary and Tertiary Structures of Pm-MEV

The secondary structure of Pm-MEV was predicted using the PSIPRED Workbench (https://bioinf.cs.ucl.ac.uk/psipred/, accessed on 10 January 2026). The tertiary structure was modeled using the Robetta server (http://robetta.bakerlab.org/, accessed on 10 January 2026). The structural quality of the tertiary model was analyzed using the ProSA-web server (https://prosa.services.came.sbg.ac.at, accessed on 11 January 2026). Validation of the constructed model was performed using the SAVES v6.1 server (https://saves.mbi.ucla.edu/, accessed on 12 January 2026), employing both the ERRAT and PROCHECK algorithms. An ERRAT overall quality factor greater than 50 was established as the criterion for a high-quality model. Furthermore, the model quality was assessed using the MolProbity server [21] (http://molprobity.biochem.duke.edu/index.php, accessed on 12 January 2026).

### 2.8. Prediction of B-Cell Epitopes for Pm-MEV

The B-cell epitopes of Pm-MEV were predicted using the ElliPro server (https://tools.iedb.org/ellipro/, accessed on 10 January 2026) with default parameters.

### 2.9. Molecular Docking of Pm-MEV with TLR2 and TLR4

The PDB files for Toll-like receptor 2(TLR2) (PDB ID: 2Z7X) and Toll-like receptor 4(TLR4) (PDB ID: 2Z63) were retrieved from the NCBI Molecular Modeling Database (MMDB) (https://www.ncbi.nlm.nih.gov/structure/, accessed on 13 January 2026). Ligand-receptor docking analysis was performed using the ClusPro 2.0 server (https://cluspro.bu.edu/login.php?redir=/home.php, accessed on 14 January 2026). The interaction interfaces of the docked complexes were analyzed using PDBePISA (https://www.ebi.ac.uk/pdbe/pisa/, accessed on 15 January 2026). The docked complexes were visualized using PyMOL (v2.5), and atomic interactions were further analyzed using Ligplot+(v2.2).

### 2.10. iMODS-Based Normal Mode Analysis

Normal mode analysis based on an elastic network model was performed on the Pm-MEV-TLR2 and Pm-MEV-TLR4 complexes using the iMODS web server (https://imods.chaconlab.org/, accessed on 16 January 2026). This server simulates feasible large-scale motions of the complexes in conformational space by calculating low-frequency normal modes and allows interactive exploration of the resulting structures, animations, and trajectories in three dimensions.

### 2.11. Immune Simulation

The C-ImmSim server (https://kraken.iac.rm.cnr.it/C-IMMSIM/index.php, accessed on 17 January 2026) was employed to predict the capacity of Pm-MEV to induce specific antibody production and various cytokines. The simulation parameters were configured as follows: random seed = 12,345, simulation volume = 50, and simulation steps = 1050. All other parameters were maintained at their default values.

### 2.12. In Silico Cloning

To ensure efficient expression, the DNA sequence of the vaccine construct was codon-optimized using the Java Codon Adaptation Tool (JCat) (http://www.jcat.de/, accessed on 18 January 2026), with *Escherichia coli* (strain K12) selected as the host organism. The optimization quality was evaluated based on the Codon Adaptation Index (CAI) and GC content, targeting an ideal CAI value of 1.0 and a GC content range of 30% to 70%. Subsequently, the optimized gene sequence was cloned in silico into the pET28a (+) expression vector between the HindIII and EcoRI restriction sites. The final construct was analyzed using SnapGene software.

## 3. Results

### 3.1. Selection of Dominant Antigen Epitopes and Construction of Pm-MEV

**Figure 2:**
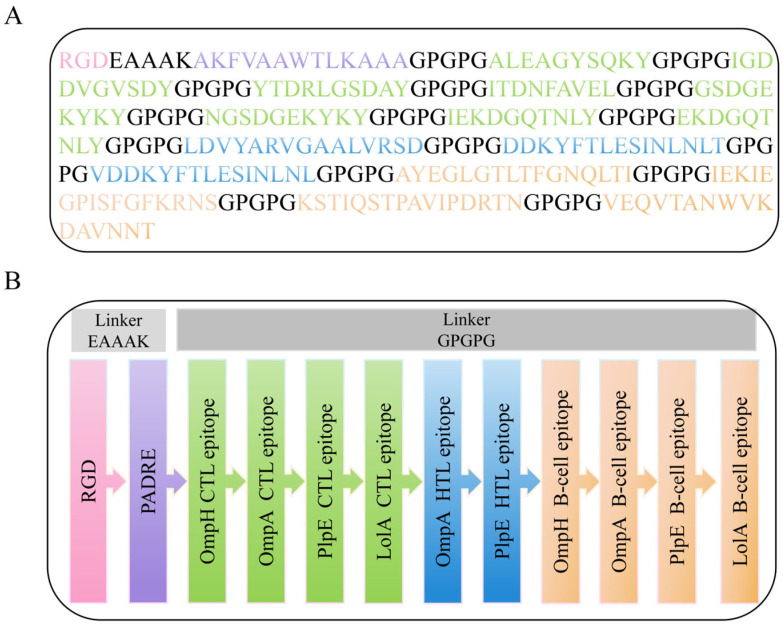
Construction of the multi-epitope-based peptide vaccine (MEV). (**A**) Amino acid sequence of the vaccine construct, highlighting CTL epitopes, HTL epitopes, B-cell epitopes, linker peptides, RGD peptide, and PADRE sequence; (**B**) Schematic diagram of the vaccine construct.

The amino acid sequences of the four dominant antigens were retrieved from the NCBI database, and their antigenicity was predicted using VaxiJen (Table 1). A total of 39 CTL epitopes (Supplementary Table S1) and 11 HTL epitopes (Supplementary Table S2) were predicted using the IEDB MHC-I and MHC-II binding servers, respectively. Following further screening via VaxiJen v2.0, 8 CTL epitopes were selected. Additionally, from the 11 HTL candidates, 3 epitopes were selected based on positive IFN-γ-inducing capability and an antigenicity score >0.5. For B-cell immunity, 16 linear epitopes were predicted using the ABCpred server (Supplementary Table S3), from which 4 were ultimately selected as candidates for Pm-MEV construction (Table 2). Finally, the selected CTL, HTL, and linear B-cell epitopes were fused using GPGPG linkers. The RGD peptide and PADRE sequence were attached via EAAAK linkers. The resulting polypeptide construct was designated as Pm-MEV; its schematic diagram and amino acid sequence are presented in Figure 2.

### 3.2. Prediction of Antigenicity, Allergenicity, and Toxicity of Pm-MEV

The immunogenicity score of Pm-MEV was predicted to be 1.7 by the IEDB MHC-I server, while the VaxiJen v2.0 server indicated an antigenicity score of 1.24 (Supplementary Table S4). Physicochemical analysis via the ProtParam server (Table 3) revealed that the construct consists of 282 amino acids with a molecular weight of approximately 28.37 kDa and a theoretical Isoelectric point (pI) of 4.58. The instability index, aliphatic index, and grand average of hydropathicity (GRAVY) were calculated to be 6.80, 59.18, and −0.591, respectively. Furthermore, SOLpro analysis predicted the protein to be soluble with a probability of 0.87, suggesting a high likelihood of soluble expression. The assessments conducted using AllerTOP v.2.0 and ToxinPred confirmed that Pm-MEV possesses neither allergenicity nor toxicity, indicating its potential safety. However, experimental verification is still required. Collectively, these results characterize Pm-MEV as a stable, hydrophilic protein with robust antigenicity, and a favorable safety profile, supporting its potential as a promising multi-epitope vaccine candidate.

### 3.3. Prediction and Analysis of Secondary and Tertiary Structures of Pm-MEV

**Figure 3:**
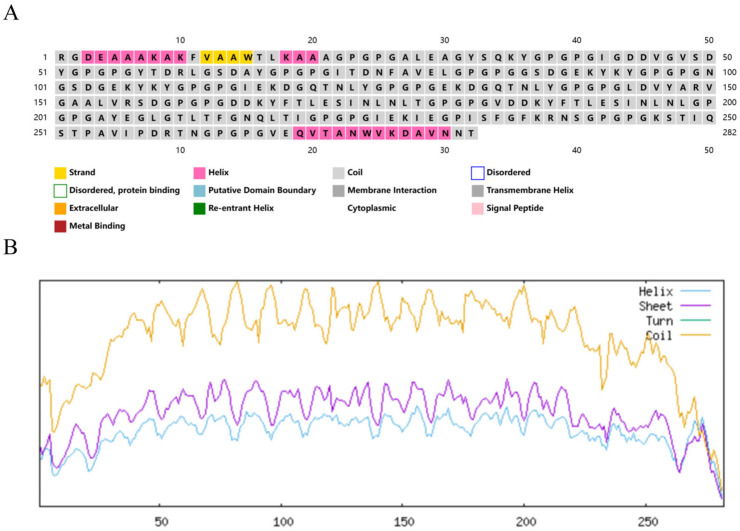
Secondary Structure Prediction of the Pm-MEV. (**A**) Secondary structure prediction of MEVs using the PSIPRED tool, the horizontal axis is the amino acid position; (**B**) Secondary structure prediction of MEVs using the SOPMA tool, the horizontal axis is the amino acid position.

**Figure 4:**
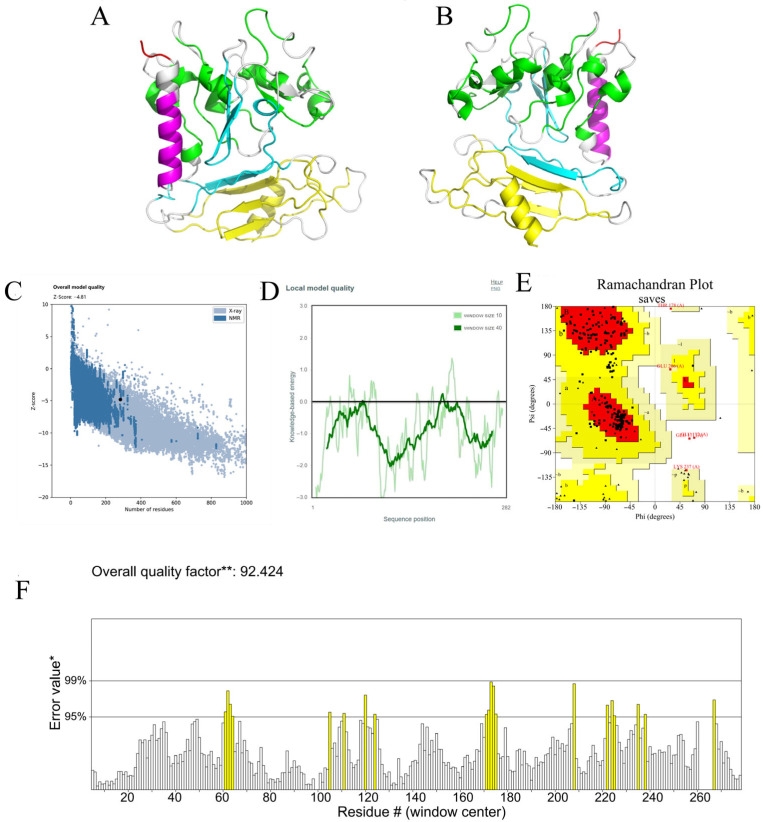
Predicted Tertiary Structure and Structural Quality Assessment of MEV. (**A**,**B**) The tertiary structure of the Pm-MEV is depicted as a cartoon representation. The tertiary structure was displayed in front view and back view. The various colors represent the positions of different amino acid sequences within the Pm-MEV tertiary structure: red indicates RGD, purple indicates PADRE, green indicates CTL epitopes, blue indicates HTL epitopes, and yellow indicates B-cell epitopes; (**C**) ProSA model quality assessment; (**D**) Local model quality; (**E**) Ramachandran diagram. The triangles represent glycine residues, and the squares represent other standard amino acid residues, with residues located in most favoured regions [A, B, L], additional allowed regions [a, b, l, p], and generously allowed regions [~a, ~b, ~l, ~p] as indicated in the figure; (**F**) ERRAT diagram. * indicates the reference error value on the ERRAT score plot used for evaluation. ** Expressed as the percentage of the protein for which the calculated error value falls below the 95% rejection limit.

Secondary structure prediction via the PSIPRED and SOPMA servers revealed that the Pm-MEV sequence is predominantly composed of random coils (98.58%), with a minor proportion of α-helices (1.42%) (Figure 3). The tertiary structure of Pm-MEV was modeled using the Robetta server and visualized using PyMOL (Figure 4A,B). Quality assessment via ProSA-web yielded a Z-score of −4.81 (Figure 4C), indicating that the model possesses overall stereochemical and energetic properties characteristic of native proteins. The local model quality plot displayed a stable energy profile fluctuating near the baseline, suggesting that the Pm-MEV structure is stable without significant regions of high energy or conflict (Figure 4D).

Ramachandran plot analysis generated by PROCHECK revealed that 81.6% of residues were located in the most favored regions, 15.7% in additionally allowed regions, 1.1% in generously allowed regions, and 1.6% in disallowed regions (Figure 4E). Additionally, the ERRAT overall quality factor was 92.42, confirming the high quality of the structural model (Figure 4F). Furthermore, the tertiary model was refined using the MolProbity server, which optimized hydrogen placement and corrected side-chain flips for Asn, Gln, and His residues. Analysis of the refined model demonstrated excellent stereochemical quality: 91.1% of residues were in the favored regions of the Ramachandran plot, with only 2.14% outliers. The MolProbity score was 1.79 (Supplementary Figure S1), and the all-atom clash score was 5.11 (Supplementary Figure S2). These metrics collectively indicate that the predicted tertiary structure of Pm-MEV is a reliable, high-confidence model suitable for subsequent molecular docking and functional mechanistic studies.

### 3.4. Prediction of B-Cell Epitopes

A total of 18 linear (continuous) B-cell epitopes were predicted using the ElliPro server (Table 4). These epitopes ranged in length from 4 to 33 residues, with scores spanning from 0.517 to 0.806. Additionally, 167 residues were identified as participating in discontinuous B-cell epitopes (Table 5), exhibiting scores ranging from 0.609 to 0.694.

### 3.5. Molecular Docking of Pm-MEV with TLRs

**Figure 5:**
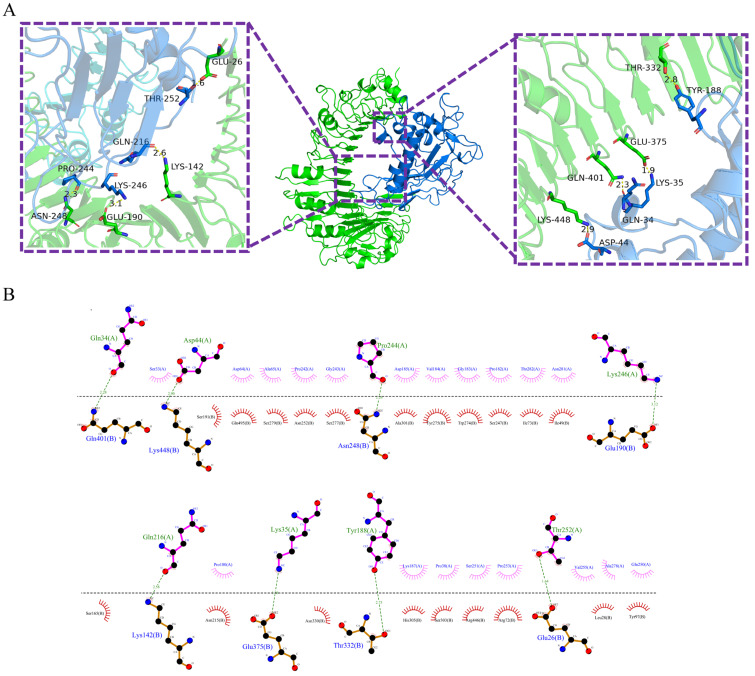
(**A**) Predicted docking complex between the TLR2 receptor (green cartoon, labeled as B chain) and the multi-epitope vaccine Pm-MEV (blue cartoon, labeled as A chain). The bar-shaped structures highlight the interacting amino acid residues at the binding interface. Hydrogen bonding interactions are depicted as green dashed lines, with specific residues involved: TLR2 residues GLN401, LYS448, ASN248, GLU190, LYS142, GLU375, THR332, and GLU26 form hydrogen bonds with Pm-MEV residues GLN34, ASP44, PRO244, LYS246, GLN216, LYS35, TYR188, and THR252; (**B**) The visualization tool Ligplot+ captured their two-dimensional images.

**Figure 6:**
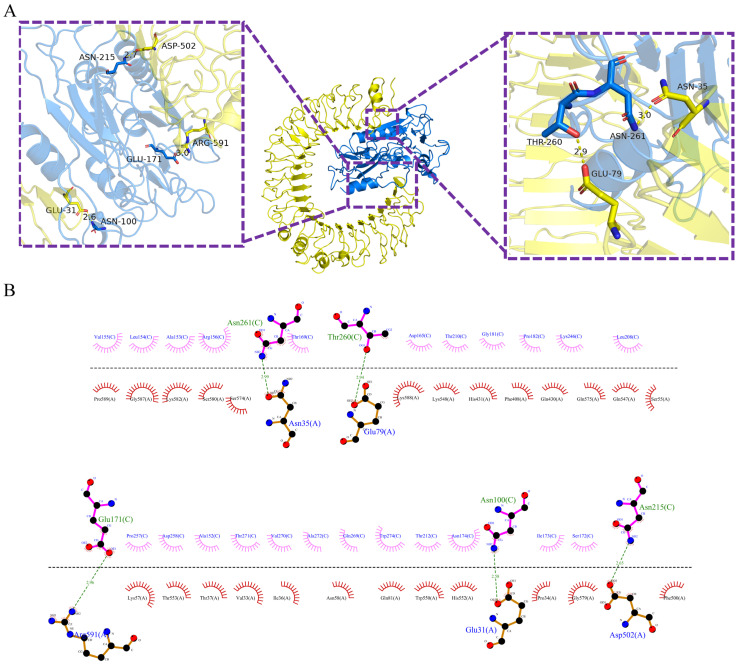
(**A**) Predicted docking complex between the TLR4 receptor (yellow cartoon, labeled as A chain) and the multi-epitope vaccine Pm-MEV (blue cartoon, labeled as C chain). The bar-shaped structures highlight the interacting amino acid residues at the binding interface. Hydrogen bonding interactions are depicted as green dashed lines, with specific residues involved: TLR4 residues ASN35, GLU79, ARG591, GLU31, and ASP502 form hydrogen bonds with Pm-MEV residues ASN261, THR260, GLU171, ASN100, and ASN215; (**B**) The visualization tool Ligplot+ captured their two-dimensional images.

To investigate the interaction characteristics and predict the final 3D complex structures, Pm-MEV was docked with TLR2 and TLR4 using the ClusPro 2.0 server. Ten models were generated for each complex, and the top-ranked model was selected as the optimal structure for further analysis. Interface analysis using the PDBePISA server revealed that the Pm-MEV-TLR2 complex is stabilized by 8 hydrogen bonds and 4 salt bridges, with a binding free energy of −4.6 kcal/mol. Similarly, the Pm-MEV-TLR4 complex exhibited 5 hydrogen bonds and 5 salt bridges, with a binding free energy of −10.1 kcal/mol (Supplementary Tables S5–S9). PyMOL software visualizes the complex docking (Figure 5A and Figure 6A). To further elucidate the specific residues involved in these binding interfaces, 2D intermolecular interaction maps were generated using LigPlot+ (Figure 5B and Figure 6B).

### 3.6. iMODS-Based Normal Mode Analysis

**Figure 7:**
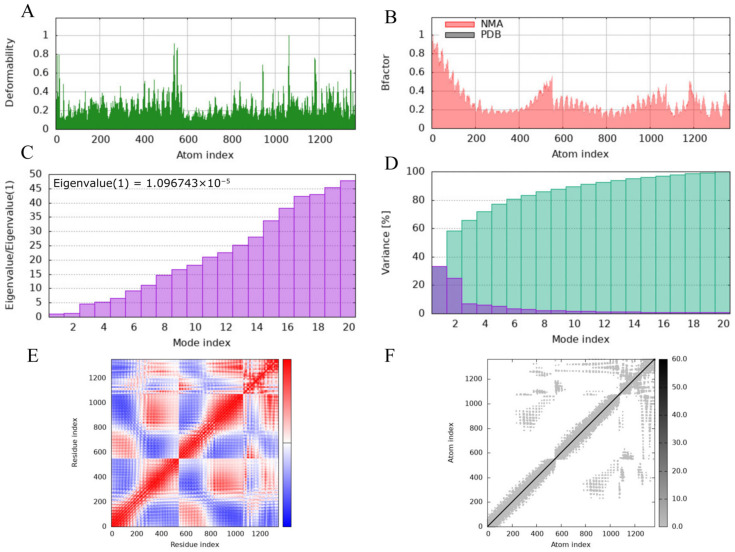
Normal mode analysis of MEV-TLR2 Complexes. (**A**) Deformability analysis plot; (**B**) B-factor analysis plot; (**C**) Eigenvalue distribution plot; (**D**) Structural variance plot. The variance associated with each normal mode is inversely related to the eigenvalue. Colored bars show the individual (purple) and cumulative (green) variance; (**E**) Covariance matrix plot; Covariance matrix indicates coupling between pairs of residues, i.e., whether they experience correlated (red), uncorrelated (white), or anti-correlated (blue) motions; (**F**) Elastic network model plot.

To assess the conformational stability and dynamic flexibility of the constructed Pm-MEV-TLR2 complex, normal mode analysis based on an elastic network model was performed. As illustrated in Figure 7, the atomic deformability (Figure 7A) and predicted B-factors (Figure 7B) exhibited highly consistent trends, collectively revealing the distribution of structural flexibility within the complex. Overall, the core regions of the complex displayed minimal fluctuations, indicating a compact and rigid structural organization. The global dynamic properties of the system were characterized by the eigenvalue spectrum (Figure 7C). The calculated eigenvalue of 1.096743 × 10^−5^ indicates that the complex resides in a local minimum of the potential energy surface, suggesting a stable topology. Variance analysis (Figure 7D) demonstrated that the first 20 low-frequency modes (particularly the first 5) accounted for over 80% of the total variance. This suggests that these collective motions dominate the functional conformational transitions of the complex. Furthermore, the covariance matrix map (Figure 7E) characterized the inter-residue dynamical correlations. Prominent red regions in the map indicated significant positive correlations among various residue groups, reflecting concerted motions. Notably, the binding interface between Pm-MEV and TLR2 exhibited a dense pattern of alternating red and blue signals. This implies the presence of complex coupled and anti-correlated motions, which are likely critical for binding affinity and specific recognition.

**Figure 8:**
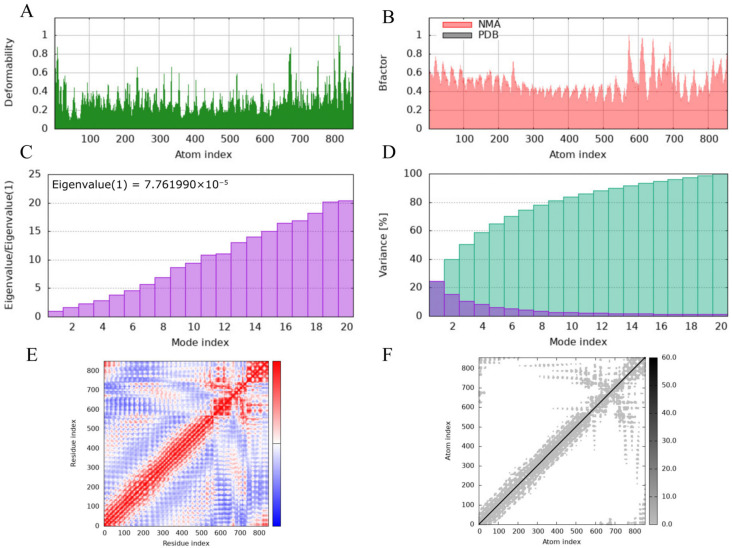
Normal mode analysis of Pm-MEV-TLR4 Complexes. (**A**) Deformability analysis plot; (**B**) B-factor analysis plot; (**C**) Eigenvalue distribution plot; (**D**) Structural variance plot; (**E**) Covariance matrix plot; (**F**) Elastic network model plot.

Elastic network analysis of the MEV-TLR4 complex indicated a relatively high overall flexibility (Figure 8F), characterized by an eigenvalue of 7.761990 × 10^−5^ (Figure 8C). The structural model demonstrated dynamic reliability, as evidenced by the high concordance between NMA-calculated B-factors and the experimental B-factors from the PDB model (Figure 8B). Deformability analysis highlighted regions of local high flexibility, while the covariance matrix map revealed complex patterns of correlated and anti-correlated motions at the binding interface (Figure 8E,F). Concurrently, the Gaussian network model indicated relatively low fluctuation within the interface core, suggesting that the binding of Pm-MEV to TLR4 induced local structural stabilization (Figure 8F). Notably, the overall flexibility of the MEV-TLR4 complex was significantly higher than that observed in the MEV-TLR2 complex.

### 3.7. Immune Simulation

**Figure 9:**
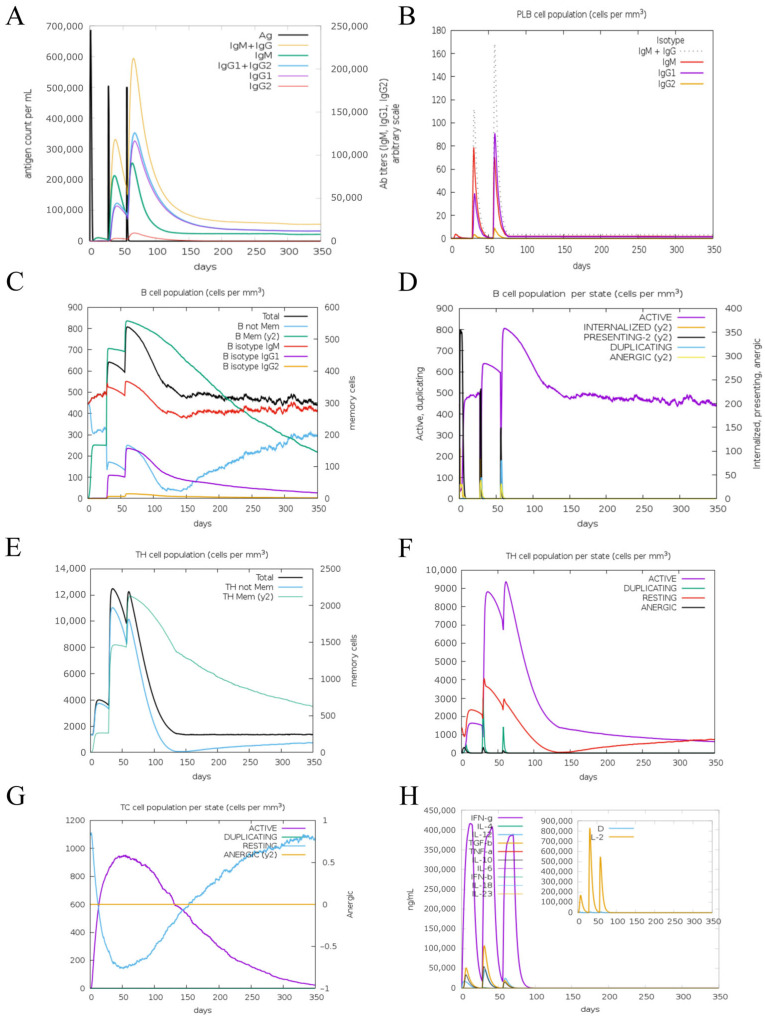
Immunological Simulation Analysis. (**A**) Immunoglobulin response to MEV; (**B**) Changes in plasma cell (PLB) populations; (**C**) B-cell populations; (**D**) Number of B cells per state; (**E**) TH cell populations; (**F**) Number of TH cells per state; (**G**) Number of TC cells per state; (**H**) Concentrations of interleukins and cytokines.

In silico immune simulation was conducted using C-ImmSim to predict the immune response in an ovine model. The results demonstrated that IgM and IgG antibody titers increased significantly following the second and third immunizations with Pm-MEV (Figure 9A). Concurrently, the population of in silico expressing IgM and IgM + IgG isotypes exhibited a marked increase after the booster doses (Figure 9B). Vaccination also induced substantial expansions in both the total B-cell count and the active B-cell population (Figure 9C,D). Additionally, Pm-MEV significantly increased the total numbers of T helper (TH) cells and active T cells (Figure 9E,F), while active cytotoxic T cells (CTLs) showed a sustained increasing trend post-vaccination (Figure 9G). Furthermore, the expression levels of interferon-gamma (IFN-γ) and interleukin-2 (IL-2) were significantly upregulated (Figure 9H). Collectively, these findings suggest that Pm-MEV is capable of eliciting a robust immune response against *P. multocida*.

### 3.8. Codon Optimization and In Silico Cloning

**Figure 10:**
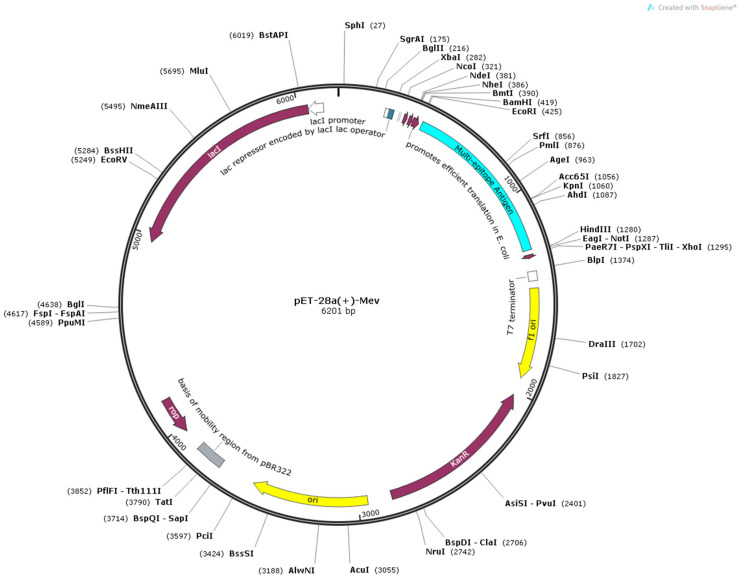
Plasmid Construction of the Codon-Optimized Vaccine in the *E. coli* K12 Expression System. The inserted DNA sequence is highlighted in blue. The fragment was inserted between HindIII and EcoRI restriction sites.

The DNA sequence of the Pm-MEV construct was codon-optimized using JCat. The optimized sequence exhibited a Codon Adaptation Index (CAI) of 0.96 and a GC content of 57.21%, which falls within the optimal range. These metrics indicate a high potential for efficient expression in the *Escherichia coli* system. Subsequently, the optimized sequence was cloned into the pET-28a (+) vector via the HindIII and EcoRI restriction sites, yielding a final recombinant construct of 6201 bp (Figure 10). While the design is theoretically optimized for bacterial expression, experimental validation is required to confirm the solubility, proper folding, and functional activity of the expressed protein.

## 4. Discussion

Pm is a significant pathogen responsible for a wide spectrum of diseases in both livestock and wildlife. Environmental stressors frequently precipitate outbreaks of pasteurellosis, resulting in substantial economic losses to the animal industry [22]. In China, control strategies primarily rely on antibiotics and vaccination. However, the prolonged application of broad-spectrum antibiotics has exacerbated the emergence of antimicrobial resistance, thereby complicating disease management. Consequently, vaccination of susceptible populations in endemic regions remains the primary intervention for the prevention and control of pasteurellosis. Current vaccine platforms against Pm primarily include inactivated, live attenuated, subunit, and DNA vaccines. Nevertheless, these conventional vaccines are often associated with limitations, such as insufficient antigenicity, poor cross-protection, and the risk of virulence reversion [23].

In contrast to traditional vaccines, which entail lengthy development cycles and high costs, MEVs offer superior biosafety. This is attributed to their ability to selectively activate specific immune responses and their production process, which eliminates the need for in vitro pathogen cultivation. Recent revolutionary advancements in information technology, molecular biology, and genomic databases have established immunoinformatics as a powerful platform for rational vaccine design. By accurately predicting immunogenic components and reconstructing antigens, this computational approach allows for the simulation and evaluation of vaccine candidates prior to experimental validation, significantly enhancing development efficiency while mitigating potential side effects [24]. This methodology not only substantially reduces research costs and timeframes but also employs multi-target and multi-mechanistic designs to synergistically elicit innate, humoral, and cellular immune responses. Consequently, MEVs can induce more comprehensive and durable protection compared to traditional monovalent vaccines, representing a highly efficient, precise, and reliable paradigm for vaccine development [25].

Epitope-based vaccines have demonstrated promising potential in immunizing against various infections, having been proven to elicit protective cellular and humoral immunity [26]. Currently, MEV development is predominantly focused on viral diseases, such as COVID-19 [27] and *Dengue virus* [28]. However, increasing research is also directed toward bacterial pathogens, including *Brucella* [9], *Escherichia coli* [4], and *Salmonella* [5]. Therefore, the development of vaccines based on epitope antigens represents a promising future direction for the control of Pm infections. A recent study evaluated sheep-derived inactivated vaccines against the Pm D serotype and recombinant OmpH vaccines. The results demonstrated that the inactivated vaccine provided 80% protection against homologous serotypes, but only 0–20% protection against heterologous serotypes (A and F), while the recombinant vaccine showed no protection against any serotypes [29]. Therefore, further exploration is needed for the development of multivalent and novel vaccines. In contrast, the multi-epitope design in this study, which incorporates four conserved antigens (OmpH; OmpA; PlpE and LolA), theoretically offers broader cross-protection, overcoming the limitations of traditional vaccines.

Using immunoinformatics approaches, we screened for highly antigenic, non-toxic, and non-allergenic candidate epitopes, selected as immunodominant epitopes based on prior research and literature support, resulting in the selection of 8 CTL, 3 HTL, and 4 B-cell epitopes. To construct an efficient multi-epitope vaccine, these epitopes were assembled in an ordered manner using a rational selection of specific linkers and adjuvants. In our construction strategy, the Arg-Gly-Asp (RGD) peptide was introduced as a molecular adjuvant, as research indicates that RGD significantly enhances antigenicity through its adjuvant effects [30,31]. Concurrently, the Pan HLA-DR-binding epitope (PADRE), capable of broad MHC class II binding, was incorporated to augment long-term immune responses [32]. Regarding epitope linkage, GPGPG sequences were employed as flexible spacers to facilitate effective epitope presentation and proteasomal processing [33]. The EAAAK rigid linker was selected to connect the adjuvant and antigen regions, aiming to maintain structural stability and minimize unintended interactions, thereby optimizing the physicochemical properties and immunological efficacy of the vaccine [34]. Through this systematic design, a multi-epitope vaccine candidate composed of 282 amino acids was ultimately generated. Physicochemical and antigenicity analyses demonstrate that the vaccine is structurally stable, highly soluble, strongly antigenic, and free of allergenic risks, exhibiting promising potential as a prophylactic vaccine against Pm infection. A limitation of the present study pertains to the MHC analysis, wherein epitope predictions were conducted using human HLA alleles rather than ovine OLA molecules. This approach was necessitated by the current unavailability of comprehensive OLA datasets in established immunoinformatic repositories such as the IEDB. Nevertheless, given the high degree of structural conservation of the MHC peptide-binding groove across mammalian species, well-characterized HLA alleles may serve as a reasonable surrogate for identifying peptides with immunogenic potential that could also be recognized by OLA molecules. This methodology is consistent with recent efforts in livestock vaccine development, as demonstrated in a study on an *Anaplasma ovis* vaccine for sheep, which similarly employed human alleles for epitope prediction due to limited OLA-specific data [35]. To minimize species-specific uncertainty, our screening strategy prioritized epitopes that are conserved across *P. multocida* strains and exhibit broad MHC binding affinity. It should be emphasized, however, that these in silico predictions require experimental validation through future OLA binding assays or in vivo immunization trials in sheep.

In this study, the physicochemical properties of Pm-MEV were systematically analyzed. Predictions via ProtParam yielded a theoretical isoelectric point (pI) of 4.58, which is significantly lower than the physiological pH. This indicates that the protein carries a net negative charge in the serum environment, which facilitates water solubility and minimizes the risk of non-specific protein aggregation [36]. The instability index was predicted to be 6.8 (<40), suggesting that the protein possesses high stability during in vitro expression, purification, and storage at 4 °C. The Grand Average of Hydropathicity (GRAVY) was −0.591 (<0), which, combined with a SOLpro probability of 0.87 (>0.5), consistently indicates that the protein is highly hydrophilic. This suggests that Pm-MEV can likely be expressed in soluble form in *Escherichia coli* or yeast systems without the need for complex refolding steps. Furthermore, structural evaluation via Ramachandran plot, ERRAT, and ProSA-web confirmed that the 3D model meets quality standards. This rational folding provides a structural basis for effectively activating B cells and inducing specific antibody responses. Collectively, Pm-MEV exhibits favorable physicochemical characteristics and structural reliability, meeting the fundamental requirements for downstream development and application.

Toll-like receptors (TLRs) serve as critical pattern recognition receptors on the surface of innate immune cells and certain non-immune cells; they recognize conserved pathogen-associated molecular patterns to initiate innate immune responses [37]. Among them, TLR2 and TLR4 play pivotal roles in recognizing bacterial components and promoting specific T-cell immunity [38]. TLR4, in particular, enhances host anti-bacterial immunity by binding to pathogen structural proteins, thereby activating downstream signaling pathways and inducing the secretion of pro-inflammatory cytokines [39]. Based on these mechanisms, molecular docking analysis was performed between Pm-MEV and both TLR2 and TLR4. The results revealed a computed binding free energy of −10.1 kcal/mol for the Pm-MEV-TLR4 interaction, with 5 hydrogen bonds and 5 salt bridges contributing to the predicted binding interface. This suggests a potentially favorable binding affinity, indicating that Pm-MEV may possess structural features compatible with engaging the TLR4 pathway, offering a possible structural basis for subsequent immune responses. Molecular docking analysis provides preliminary structural insights into the potential interaction modes between Pm-MEV and TLRs, proposing a hypothetical recognition basis that requires subsequent confirmation through cytological experiments and functional validation.

This study employed normal mode analysis using the iMODS server to evaluate the structural stability of the vaccine. It is important to acknowledge that this approach represents a limitation, as iMODS does not provide the detailed trajectory data (e.g., RMSD, RMSF, and binding energy calculations) generated by conventional all-atom molecular dynamics (MD) simulations such as GROMACS or AMBER. Nevertheless, the use of iMODS is considered reasonable in the context of this preliminary computational study. iMODS offers a computationally efficient alternative for assessing the collective motion patterns of macromolecular complexes and provides key parameters including deformability, B-factor, eigenvalues, and covariance plots, which collectively offer insights into complex stability. Moreover, this method has been widely adopted in recent vaccine design and immunoinformatics studies as an effective preliminary screening tool [40,41], supporting its appropriateness for the current scope of work.

Immunological prediction analysis indicates that Pm-MEV can effectively stimulate both innate and adaptive immune responses in the ovine model. The simulation results demonstrate that vaccination could induce high levels of cytotoxic T lymphocytes (CTLs), helper T lymphocytes (HTLs), memory cells, and specific antibodies (Ig). Concurrently, the levels of critical cytokines, such as interferon-γ (IFN-γ) and interleukin-2 (IL-2), were significantly elevated. Notably, IFN-γ plays a pivotal role in antibacterial immunity by activating macrophages to eliminate intracellular pathogens [42]. In summary, Pm-MEV is capable of mobilizing diverse immune cells and cytokines, demonstrating the potential to elicit a comprehensive immune response.

Furthermore, in silico cloning analysis confirmed the feasibility of high-level expression of the vaccine construct in the *Escherichia coli* system, establishing a basis for subsequent large-scale production. Nevertheless, these predictive results require further validation through in vivo experiments to definitively ascertain actual protective efficacy and safety.

Notwithstanding these limitations, this study offers several contributions. To our knowledge, this is the first immunoinformatics-based design of a MEV specifically targeting *Pasteurella multocida* strains affecting sheep. By focusing on the dominant antigens OmpH; OmpA; PlpE and LolA, we identified a set of candidate CTL, HTL, and B-cell epitopes with favorable antigenicity and safety profiles. The final vaccine construct was designed with consideration of structural stability and immunogenic potential. Although preliminary, this work provides a basis for further experimental evaluation, including in vitro binding assays and in vivo immunization studies in sheep, to assess the actual protective efficacy of the proposed vaccine candidate.

## 5. Conclusions

This study, a MEV against Pm, was successfully designed utilizing reverse vaccinology and immunoinformatics strategies. The vaccine exhibits high antigenicity and a favorable safety profile, is capable of stable binding to TLR4, and is predicted to induce potent and durable humoral and cellular immune responses in the host. These findings provide a design basis and theoretical framework for the rapid development of Pm vaccines. It is important to note, however, that the conclusions presented herein are based on computational simulations; the actual immunoprotective efficacy and safety profile await further verification through subsequent in vitro and in vivo experiments.

## Figures and Tables

**Table 1:** Dominant Antigen Information.

Protein Name	Sequence	Vaxijen Score	Antigenicity
OmpH AAL25842.1	SSNLANAATVYNQDGTKVDVNGSVRLLLKKEKDERGDLVDNGSRVSFKASHDLGEGLSALAYAELRFSTKVKKTVKEGQVERTYEVERIGNDVHVKRLYAGFAYEGLGTLTFGNQLTIGDDVGVSDYTYFLGGINNLLSSGEKAINFKSAEFNGFTFGGAYVFSADADKQAPRDGRGFVVAGLYNRKMGDVGFALEAGYSQKYVTVAKQEKEKAFMVGTELSYAGLALGVDYAQSKVTNVEGKNVLLKWLNYDIMIRQSSLIHLAKKPKGTPESSICRRLNFNSETCEGWEKNLCYTKMCGWSALYLSLKRVPVL	0.6103	ANTIGEN
OmpA AFS89615.1	MKKTAIALTIAALAAASVAQAAPQPNTFYVGAKAGWASFHDGLNQIEKIEGPISFGFKRNSVTYGVFGGYQITDNFAVELGYDDFGRAKVRATDPKTKETVGEAKHTNHGAHLSLKASYPVLDGLDVYARVGAALVRSDYKVYDKELADLSFLKRTHSTQVSPVFAGGLEYAFMPELALRVEYQWLNNVGKLKDAKGERVDYRPDIGSVTAGLSYRFGQSVYVPEVVSKTFTLNSDVTFGFDKADLKPAAQNVLDGIYGEIAQLKSASVAVARYTDRLGSDAYNLKLSQRRADTVANYLVAKGVAQNAISATGHGEANPVTGNKCDSVKGRKALIACLADDRRVEIAVKGNK	0.6863	ANTIGEN
PlpE PNQ01671.1	MKYFDTKKSLPVLCSLLITACSGGGGGNNNVPHPPVEKRTVATTQQVAAKPTPPSESLVKRVLNNSENNPPSRESEKKASQTPPQTDSAKSTIQSTPAVIPDRTNSLTSKKGQIQMSPEWIGKEEKKYAQYSWEHTPESIPVFKLIENNQYKYVDDKYFTLESINLNLTKENQVKEGSYQFSLLDSGVYYGHYLSSNDGIHPEYNFVIAFDKNREYTLKDITAEYYNSEGFNYAISDRMKGDYIWQVGDVRLFYTNGSVHGEIVEVNDGSKTALFRFENTADRNPNQIVIVPERDNRHGLSPRGDRMIMDMHFINGSDGEKYKYVVGHGNSDRYYGTLFATKKDKE	0.6803	ANTIGEN
LolA AAK02340.1	MNNMLLKTTALLTLFLSSAAWADAASELQQRLSKVNVLSADYAQTVSSSDGKNVQQGSGTLKIKRPNLFRMDNKTPQENQIISDGKTLWFYDPFVEQVTANWVKDAVNNTPFVLLTSDDSSHWAQYNVEQKADTFTLKPKAKQSNIKQFDIRIDSEGVLRNFSTIEKDGQTNLYILRNITNQPLADGIFKFSVPKGVELDDQRQK	0.5391	ANTIGEN

**Table 2:** Candidate epitopes of dominant antigens.

Protein Name	CTL Epitope	HTL Epitope	B-Cell Epitope
OmpH	ALEAGYSQKY	~	AYEGLGTLTFGNQLTI
	IGDDVGVSDY		
OmpA	YTDRLGSDAY	LDVYARVGAALVRSD	IEKIEGPISFGFKRNS
	ITDNFAVEL		
PlpE	GSDGEKYKY	DDKYFTLESINLNLT	KSTIQSTPAVIPDRTN
	NGSDGEKYKY	VDDKYFTLESINLNL	
LolA	IEKDGQTNLY	~	VEQVTANWVKDAVNNT

**Table 3:** Evaluation of Physicochemical Properties, Antigenicity, Allergenicity, and Toxicity of the Pm-MEV.

Number of Amino Acids	Molecular Weight	Theoretical pI	Instability Index	Aliphatic Index	GRAVY	SOLUBLE	Toxicity	Allergenicity
282	28,371.10 Da	4.58	6.80	59.18	−0.591	0.87	Non-toxic	non-allergenic

**Table 4:** Continuous B-cell epitope residues and scoring.

No	Chain	Start	End	Peptide	Number of Residues	Score
1	A	237	269	KRNSGPGPGKSTIQSTPAVIPDRTNGPGPGVEQ	33	0.806
2	A	1	6	RGDEAA	6	0.772
3	A	178	189	TGPGPGVDDKYF	12	0.759
4	A	96	121	PGPGNGSDGEKYKYGPGPGIEKDGQT	26	0.697
5	A	32	41	YSQKYGPGPG	10	0.688
6	A	127	133	GPGEKDG	7	0.674
7	A	65	74	AYGPGPGITD	10	0.65
8	A	156	165	RSDGPGPGDD	10	0.645
9	A	219	228	IGPGPGIEKI	10	0.63
10	A	140	144	PGPGL	5	0.559
11	A	214	217	GNQL	4	0.544
12	A	200	209	PGPGAYEGLG	10	0.517

**Table 5:** Discontinuous B-cell epitope residues and scores.

No	Residues	Number of Residues	Score
1	A:G2, A:D3, A:E4, A:A5, A:A6, A:A9, A:K10	7	0.694
2	A:A13, A:L17, A:A20, A:A21, A:G22, A:P23, A:T178, A:G179, A:P180, A:G181, A:P182, A:G183, A:V184, A:D185, A:D186, A:K187, A:Y188, A:F189, A:T190, A:P200, A:G201, A:P202, A:G203, A:A204, A:Y205, A:E206, A:G207, A:L208, A:G209, A:T210, A:Q216, A:L217, A:I219, A:G220, A:P221, A:G222, A:P223, A:G224, A:I225, A:E226, A:K227, A:I228, A:E229, A:F236, A:K237, A:R238, A:N239, A:S240, A:G241, A:P242, A:G243, A:P244, A:G245, A:K246, A:S247, A:T248, A:I249, A:Q250, A:S251, A:T252, A:P253, A:A254, A:V255, A:I256, A:P257, A:D258, A:R259, A:T260, A:N261, A:G262, A:P263, A:G264, A:P265, A:G266, A:V267, A:E268, A:T271, A:N273, A:W274, A:D277, A:A278, A:V279, A:N280, A:N281, A:T282	85	0.693
3	A:Y109, A:G110, A:P111, A:G112, A:P113, A:G114, A:I115, A:E116, A:K117, A:D118, A:G119, A:Q120, A:T121, A:N122	14	0.677
4	A:D88, A:K91, A:G95, A:P96, A:G97, A:P98, A:G99, A:N100, A:G101, A:S102, A:D103, A:G104, A:E105, A:Y107, A:K108	15	0.667
5	A:G31, A:S33, A:Q34, A:K35, A:Y36, A:G37, A:P38, A:G39, A:P40, A:G41, A:D44, A:A65, A:Y66, A:G67, A:P68, A:G69, A:P70, A:G71, A:I72, A:T73, A:D74	21	0.663
6	A:G127, A:P128, A:G129, A:E130, A:K131, A:D132, A:G133, A:Q134, A:G139, A:P140, A:G141, A:P142, A:G143, A:L144, A:D145, A:R156, A:D158, A:G159, A:P160, A:G161, A:P162, A:G163, A:D164, A:D165, A:Y167	25	0.609

## Data Availability

The original contributions presented in this study are included in the article. Further inquiries can be directed to the corresponding author.

## Supplementary Materials

The following supporting information can be downloaded at: https://www.mdpi.com/article/10.3390/microorganisms14030656/s1. Figure S1: MolProbity data summary; Figure S2: MolProbity Ramachandran analysis; Table S1: B-cell epitope screening results table; Table S2: CTL epitope screening result table; Table S3: HTL epitope screening result table; Table S4: Antigenicity and Immunogenicity of Pm-MEV. Table S5: Interaction interfaces of the docked complexes. Table S6: MEV-TRL2 complex hydrogen bond statistics table. Table S7: MEV-TRL2 complex Salt bridges statistics table. Table S8: MEV-TRL4 complex hydrogen bond statistics table. Table S9: MEV-TRL4 complex Salt bridges statistics table.
