# Supplementary material for: Design of a Multi-Epitope Vaccine Against Ovine Pasteurella multocida Using Immunoinformatics Strategies
Source: Microorganisms. 2026 Mar 13;14(3):656. doi: 10.3390/microorganisms14030656 (PMC13029743; doi:10.3390/microorganisms14030656)
Supplement: Supplementary file 1 [file microorganisms-14-00656-s001.zip › microorganisms-4165611-supplementary.pdf]

Supplementary Table S1. B-cell epitope screening results table.

Protein name	Sequence	Start position	ABCpred Prediction Server score	B-cell epitope
OmpH	AYEGLGTLTFGNQLTI	103	0.94	√
	KGTPESSICRRLNFNS	269	0.92	
	CEGWEKNLCYTKMCGW	287	0.9	
	GFTFGGAYVFSADADK	154	0.88	
OmpA	IEKIEGPISFGFKRNS	46	0.98	√
	TGHGEANPVTGNKCDS	312	0.97	
	ALVRSDYKVYDKELAD	134	0.93	
	GKLKDAKGERVDYRPD	190	0.88	
PlpE	KSTIQSTPAVIPDRTN	90	0.95	√
	SLLITACSGGGGGNNN	15	0.94	
	GVYYGHYLSSNDGIHP	187	0.91	
	KGQIQMSPEWIGKEEK	111	0.9	
	GKEEKKYAQYSWEHTP	122	0.88	
LolA	VEQVTANWVKDAVNNT	95	0.89	√
	GKTLWFYDPFVEQVTA	85	0.88	
	TLKIKRPNLFRMDNKT	60	0.88	

Supplementary Table S2. CTL epitope screening result table.

Protein name	Allele	Start	End	Length	Peptide	Score	IEDB Class I-percentile rank<0.5	VaxiJen -Antigen icity＞ 0.5	CTL epitope
OmpH	HLA-A*01:01	194	203	10	ALEAGYSQKY	0.63672	0.12	0.9079	√
	HLA-A*01:01	118	127	10	IGDDVGVSDY	0.586132	0.14	1.437	√
	HLA-A*01:01	215	223	9	FMVGTELSY	0.347474	0.31	0.266	
	HLA-A*01:01	3	11	9	NLANAATVY	0.267993	0.42	0.0817	
OmpA	HLA-A*01:01	274	283	10	YTDRLGSDAY	0.993238	0.01	0.9846	√
	HLA-A*01:01	20	29	10	QAAPQPNTFY	0.591208	0.14	0.0637	
	HLA-A*01:01	72	80	9	ITDNFAVEL	0.498916	0.19	1.4867	√
	HLA-A*01:01	207	215	9	GSVTAGLSY	0.479276	0.2	0.9548	
	HLA-A*01:01	21	29	9	AAPQPNTFY	0.436207	0.24	-0.0604	
	HLA-A*01:01	265	274	10	KSASVAVARY	0.371149	0.29	0.7297	
	HLA-A*01:01	266	274	9	SASVAVARY	0.365901	0.29	0.7855	
	HLA-A*01:01	62	70	9	VTYGVFGGY	0.304694	0.37	-0.5859	
	HLA-A*01:01	163	171	9	PVFAGGLEY	0.294309	0.38	0.4005	
	HLA-A*01:01	213	222	10	LSYRFGQSVY	0.266736	0.43	1.2276	
	HLA-A*01:01	162	171	10	SPVFAGGLEY	0.266343	0.43	0.3667	
PlpE	HLA-A*01:01	195	204	10	SSNDGIHPEY	0.959604	0.01	1.1904	
	HLA-A*01:01	316	324	9	GSDGEKYKY	0.94839	0.02	2.5197	√
	HLA-A*01:01	315	324	10	NGSDGEKYKY	0.893366	0.04	2.6155	√
	HLA-A*01:01	145	153	9	LIENNQYKY	0.844146	0.05	0.1869	
	HLA-A*01:01	217	226	10	TLKDITAEYY	0.825464	0.06	0.5221	
	HLA-A*01:01	216	225	10	YTLKDITAEY	0.780739	0.07	0.5238	
	HLA-A*01:01	235	243	9	ISDRMKGDY	0.774307	0.07	0.7337	
	HLA-A*01:01	196	204	9	SNDGIHPEY	0.748172	0.08	0.9969	

	HLA-A*01:01	181	190	10	FSLLDSGVYY	0.642121	0.12	-0.1315	
	HLA-A*01:01	246	254	9	QVGDVRLFY	0.599359	0.14	1.1119	
	HLA-A*01:01	182	190	9	SLLDSGVYY	0.499701	0.19	-0.5143	
	HLA-A*01:01	153	161	9	YVDDKYFTL	0.496103	0.19	0.3099	
	HLA-A*01:01	181	189	9	FSLLDSGVY	0.454385	0.22	-0.0399	
	HLA-A*01:01	217	225	9	TLKDITAEY	0.446358	0.23	0.5136	
	HLA-A*01:01	144	153	10	KLIENNQYKY	0.400416	0.27	0.2041	
	HLA-A*01:01	313	322	10	FINGSDGEKY	0.392203	0.28	1.5505	
	HLA-A*01:01	330	339	10	NSDRYYGTLF	0.305603	0.37	-0.255	
	HLA-A*01:01	234	243	10	AISDRMKGDY	0.271172	0.42	0.7918	
	HLA-A*01:01	208	216	9	IAFDKNREY	0.255036	0.44	-0.8025	
	HLA-A*01:01	82	91	10	ISDGKTLWFY	0.984085	0.01	0.1675	
	HLA-A*01:01	82	90	9	ISDGKTLWF	0.626293	0.12	-0.3732	
LolA	HLA-A*01:01	117	126	10	SDDSSHWAQY	0.56039	0.16	-0.4278	
	HLA-A*01:01	165	174	10	IEKDGQTNLY	0.323269	0.34	0.7409	√
	HLA-A*01:01	166	174	9	EKDGQTNLY	0.241945	0.47	1.4155	√

Supplementary Table S3. HTL epitope screening result table.

Protein name	Allele	Start	End	Length	Peptide Sequence	score	IEDB Class II-percentile rank＜0.5	VaxiJen -Antigenicity＞0.5	Inducibility of IFN-γ epitopes	HTL epitope
OmpH	HLA-DRB1*01:01	229	243	15	GVDYAQSKVTNVEGK	0.5814	2.7	~	~	
	HLA-DRB1*01:01	7	21	15	AATVYNQDGTKVDVN	0.4735	3.5			
OmpA	HLA-DRB1*01:01	7	21	15	ALTIAALAAASVAQA	0.947	0.23	0.49	~	
	HLA-DRB1*01:01	258	272	15	YGEIAQLKSASVAVA	0.9426	0.26	0.4		
	HLA-DRB1*01:01	125	139	15	LDVYARVGAALVRSD	0.9257	0.32	0.68	POSITIVE	√
	HLA-DRB1*01:01	6	20	15	IALTIAALAAASVAQ	0.9201	0.35	0.61	~	
	HLA-DRB1*01:01	257	271	15	IYGEIAQLKSASVAV	0.9116	0.4	0.52		
PlpE	HLA-DRB1*01:01	155	169	15	DDKYFTLESINLNLT	0.9306	0.3	0.84	POSITIVE	√
	HLA-DRB1*01:01	154	168	15	VDDKYFTLESINLNL	0.8992	0.48	1.09	POSITIVE	√
LolA	HLA-DRB1*01:01	108	122	15	NNTPFVLLTSDDSSH	0.7925	1.1	~	~	
	HLA-DRB1*01:01	109	123	15	NTPFVLLTSDDSSHW	0.7473	1.4			

Supplementary Table S4. Antigenicity and Immunogenicity of Pm-MEV.

Peptide	Length	score	VaxiJen -Antigenicity
RGDEAAAKAKFVAAWTLKAAAGPGPGALEAGYSQKYGPGPGIGDDVGVSDYGPGPGYT-DRLGSDAYGPGPGITDNFA-VELGPGPGGSDGEKYKYGPGPGNGSDGEKYKYGPGPGIEKDGQTNLYGPGPGEKDGQTN-LYGPGPGLDVYARVGAALVRSDGPGPGDDKYFTLESINLNLTGPGPGVDDKYFT-LESINLNLGPGPGAYEGLGTLTFGNQLTIGPGPGIEKIEG-PISFGFKRNSGPGPGKSTIQSTPAVIPDRTNGPGPGVEQVTANWVKDAVNNT	282	1.78	1.24

Supplementary Table S5. Interaction interfaces of the docked complexes.

Molecular docking of Pm-MEV with TLRs	Binding free energy (kcal/mol)	Hydrogen bonds	Salt bridges
MEV-TRL2	-4.6	8	4
MEV-TRL4	-10.1	5	5

Supplementary Table S6. MEV-TRL2 complex hydrogen bond statistics table.

Number of Hydrogen bonds	Pm-MEV	Dist	TRL2
1	A:GLN 34	2.3	B:GLN 401
2	A:ASP 44	2.9	B:LYS 448
3	A:PRO 244	2.3	B:ASN 248
4	A:LYS 246	3.1	B:GLU 190
5	A:GLN 216	2.6	B:LYS 142
6	A:LYS 35	1.9	B:GLU 375
7	A:TYR 188	2.8	B:THR 332
8	A:THR 252	1.6	B:GLU 26

Supplementary Table S7. MEV-TRL2 complex Salt bridges statistics table.

Number of Salt bridges	Pm-MEV	Dist	TRL2
1	A:LYS 35	1.9	B:GLU 375
2	A:LYS 246	3.1	B:GLU 190
3	A:ASP 44	2.9	B:LYS 448
4	A:ASP 44	3.84	B:LYS 448

Supplementary Table S8. MEV-TRL4 complex hydrogen bond statistics table.

Number of Hydrogen bonds	Pm-MEV	Dist	TRL4
1	A:ASN 35	3	C:ASN 261
2	A:GLU 79	2.9	C:THR 260
3	A:ARG 591	3	C:GLU 171
4	A:GLU 31	2.6	C:ASN 100
5	A:ASP 502	2.7	C:ASN 215

Supplementary Table S9. MEV-TRL4 complex Salt bridges statistics table.

Number of Salt bridges	Pm-MEV	Dist	TRL4
1	A:ARG 591	3.7	C:GLU 171
2	A:ARG 591	3.2	C:GLU 171
3	A:ARG 591	3	C:GLU 171
4	A:ARG 591	3.8	C:GLU 171
5	A:ARG 591	3.2	C:GLU 171

Summary statistics

All-Atom Contacts	Clashscore, all atoms:	5.11		93rd percentile* (N=1784, all resolutions)
	Clashscore is the number of serious steric overlaps (> 0.4 Å) per 1000 atoms.			
Protein Geometry	Poor rotamers	1	0.50%	Goal: <0.3%
	Favored rotamers	197	98.50%	Goal: >98%
	Ramachandran outliers	6	2.14%	Goal: <0.05%
	Ramachandran favored	255	91.07%	Goal: >98%
	Rama distribution Z-score	-2.26 ± 0.45		Goal: abs(Z score) < 2
	MolProbity score^	1.79		86th percentile* (N=27675, 0Å - 99Å)
	Cβ deviations >0.25Å	0	0.00%	Goal: 0
	Bad bonds:	0 / 2067	0.00%	Goal: 0%
	Bad angles:	4 / 2818	0.14%	Goal: <0.1%
Peptide Omegas	Cis Prolines:	0 / 33	0.00%	Expected: ≤1 per chain, or ≤5%
Low-resolution Criteria	CaBLAM outliers	24	8.6%	Goal: <1.0%
	CA Geometry outliers	8	2.88%	Goal: <0.5%
Additional validations	Chiral volume outliers	0/277		
	Waters with clashes	0/0	0.00%	See UnDowser table for details

In the two column results, the left column gives the raw count, right column gives the percentage.
* 100th percentile is the best among structures of comparable resolution; 0th percentile is the worst. For clashscore the comparative set of structures was selected in 2004, for MolProbity score in 2006.
^ MolProbity score combines the clashscore, rotamer, and Ramachandran evaluations into a single score, normalized to be on the same scale as X-ray resolution.
Key to table colors and cutoffs here:

Supplementary Figure S1. MolProbity data summary.

MolProbity Ramachandran analysis

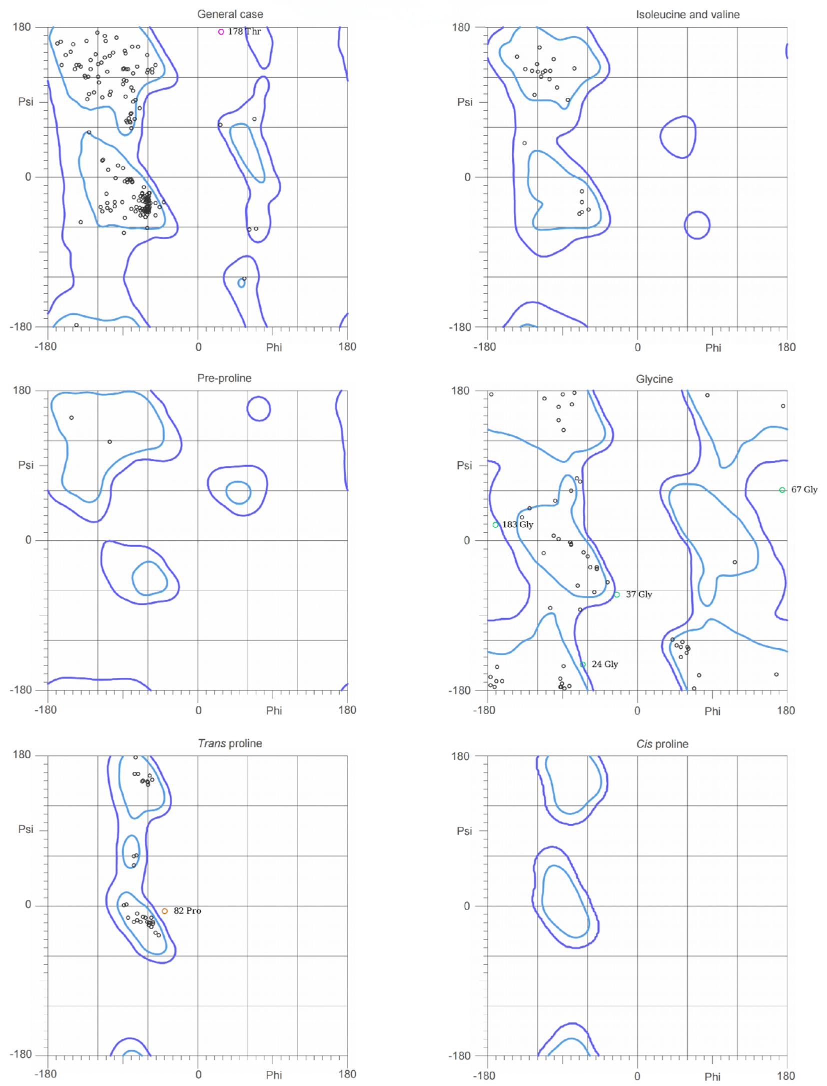

91.1% (255/280) of all residues were in favored (98%) regions.
97.9% (274/280) of all residues were in allowed (>99.8%) regions.

There were 6 outliers (phi, psi):
24 Gly (-66.0, -149.4)
37 Gly (-25.7, -65.8)
67 Gly (175.0, 61.6)
82 Pro (-40.8, -6.6)
178 Thr (28.5, 175.8)
183 Gly (-171.6, 19.4)

http://kinemage.biochem.duke.edu   Lovell, Davis, et al. Proteins 50:437 (2003)

Supplementary Figure S2. MolProbity Ramachandran analysis.

Reference

1. Lovell, S.C.; Davis, I.W.; Arendall, W.B., 3rd; de Bakker, P.I.; Word, J.M.; Prisant, M.G.; Richardson, J.S.; Richardson, D.C. Structure validation by Calpha geometry: phi, psi and Cbeta deviation. *Proteins* **2003**, *50*, 437.
